# MoS_2_@rGO Nanoflakes as High Performance Anode Materials in Sodium Ion Batteries

**DOI:** 10.1038/s41598-017-08341-y

**Published:** 2017-08-11

**Authors:** Ruxing Wang, Shu Gao, Kangli Wang, Min Zhou, Shijie Cheng, Kai Jiang

**Affiliations:** 1 State Key Laboratory of Materials Processing and Die & Mould Technology, School of Materials Science and Engineering, Huazhong University of Science and Technology, Wuhan, Hubei 430074 China; 2 State Key Laboratory of Advanced Electromagnetic Engineering and Technology, School of Electrical and Electronic Engineering, Huazhong University of Science and Technology, Wuhan, Hubei 430074 China

## Abstract

A simple one-pot hydrothermal method is developed for fabrication of MoS_2_@rGO nanoflakes using the economical MoO_3_ as the molybdenum source. Benefiting from the unique nanoarchitecture, high MoS_2_ loading (90.3 wt%) and the expanded interlayer spacing, the as-prepared MoS_2_@rGO nanoflakes exhibit greatly enhanced sodium storage performances including a high reversible specific capacity of 441 mAh g^−1^ at a current density of 0.2 A g^−1^, high rate capability, and excellent capacity retention of 93.2% after 300 cycles.

## Introduction

Rechargeable sodium ion batteries (SIBs) are seen as promising options for large scale energy storage systems because of the natural abundance of sodium and lower production cost^1–6^. Na and Li elements share many similar physical and chemical properties, which makes the strategies applied in lithium ion batteries (LIBs) valuable references to develop SIBs^7–9^. However, the large radius of Na ion and sluggish transport kinetics limit the stability and rate performance in most electrode materials and thus hinder the development of SIBs^10–14^.

Molybdenum disulfide (MoS_2_), with a well-defined lamellar structure similar to graphite, has been actively studied as anode material for both LIBs and SIBs^15–20^. Although the discharge (Na intercalation) potential is slightly higher than other anode materials like carbon and metal oxides, MoS_2_ is particularly attractive as a high capacity sodium host (theoretical capacity ~670 mA h g^−1^, based on 4 mol of Na^+^ insertion), due to its dichalcogenide structure and large interlayer spacing. However, because of its high surface energy and interlayer van der Waals attractions, the two-dimensional (2D) layered structure is thermally unstable, and has a trend to restack irreversibly during the cycling process, leading rapid capacity fading and poor rate performance^21–24^. This phenomenon is especially severe in Na^+^ storage because Na ions are about 55% larger in radius than Li ions^8, 11^. Previous studies have revealed that when MoS_2_ is cycled to very low potential (0 V versus Na/Na^+^) to achieve high capacity for SIBs, Mo metal nanoparticles are formed within a Na_2_S matrix, and subsequent electrochemical cycling operates as a sodium–sulfur redox couple. It indicates that the parent crystalline atomic structure of MoS_2_ is completely destroyed and does not reform after the Na is removed, resulting in poor cycling stability especially at high rates^12, 25–27^. Cycled in a limited voltage region (between 0.5–2.6 V versus Na/Na^+^) without destroying the 2D atomic layered structure of MoS_2_ has been proved to be an effective way to improve the cyclability, but the achieved capacity is much lower than the target^28, 29^.

To enhance the structure stability of MoS_2_, various strategies have been proposed and can be mainly divided into two categories: designing nanoarchitecture and incorporating with carbonaceous materials^15–17, 30^. Different types of nanoscale architectures have been suggested, such as nanotubes, nanoboxes, nanoflowers and hollow nanospheres, which are expected to improve the reversible capacity of MoS_2_ by alleviating the restacking of the nanosheets. For example, Hu *et al*. have fabricated MoS_2_ nanoflowers with expanded interlayers, which deliver a reversible capacity of 350 mAhg^−1^ at a limited voltage region (0.4–3 V vs. Na/Na+)^29^. Su *et al*. reported an ultrathin MoS_2_ exfoliated nanosheets, which exhibited a high initial discharge capacity of 998 mAh g^−1^ while after 100 cycles the remained discharge capacity is just 386 mAh g^−1^ ^10^. The cycle stability of nanoarchitecture MoS_2_ is still far from an ideal and exhibits an obvious capacity decay from the very beginning. The poor performance may be attributed to the large volume change during cycle which tends to severely damage the carefully designed nanostructure. Incorporating with carbonaceous materials (e.g. graphene, carbon nanotubes, and carbon coating) is another effective approach. Graphene oxide (GO) with a well-defined lamellar structure similarity to MoS_2_ may sever as an excellent carbon host to modify structural stability, improve the electronic conductivity and trigger enhanced reaction kinetics of MoS_2_. Qin *et al*. prepared MoS_2_-reduced graphene oxide (rGO) composites via a facile microwave assisted method, achieving a stable reversible capacity of 305 mAh g^−1^ at 100 mA g^−1^. However, the remained capacity was only 218 mAh g^−1^ after 50 cycles^31^. Xie *et al*. prepared MoS_2_/rGO nanocomposites with intimate two-dimensional heterointerfaces by hydrothermal method, the composite exhibits much higher initial capacity of 702 mAh g^−1^ at a current density of 20 mA g^−1^, but the capacity retention is only 49% after 100 cycles^21^. It is assumed that a suitable rGO host can optimize the electrochemical performance by improve the electron and ion transfer ability of the composite and buffer the large volume change during cycle, but in most cases the synthesis processes are relatively complicated and the molybdenum sources are rather expensive. Therefore, it is desirable to develop a MoS_2_ anode with both high capacity and long cycle life in an economical way.

Herein, we report a facile one-pot hydrothermal strategy to prepare the MoS_2_@rGO composites using an economical molybdenum source of MoO_3_. By controlling the concentration of aqueous rGO solution in hydrothermal treatment and engineering the ionic strength of precursors, three-dimensional (3D) architectures constructed of MoS_2_/rGO nanoflake is developed. The prepared MoS_2_@rGO composites with high loading of MoS_2_ exhibit excellent cycle stability and rate performance, making the synthesized MoS_2_@rGO a promising anode material for SIBs.

## Results and Discussion

**Figure 1:**
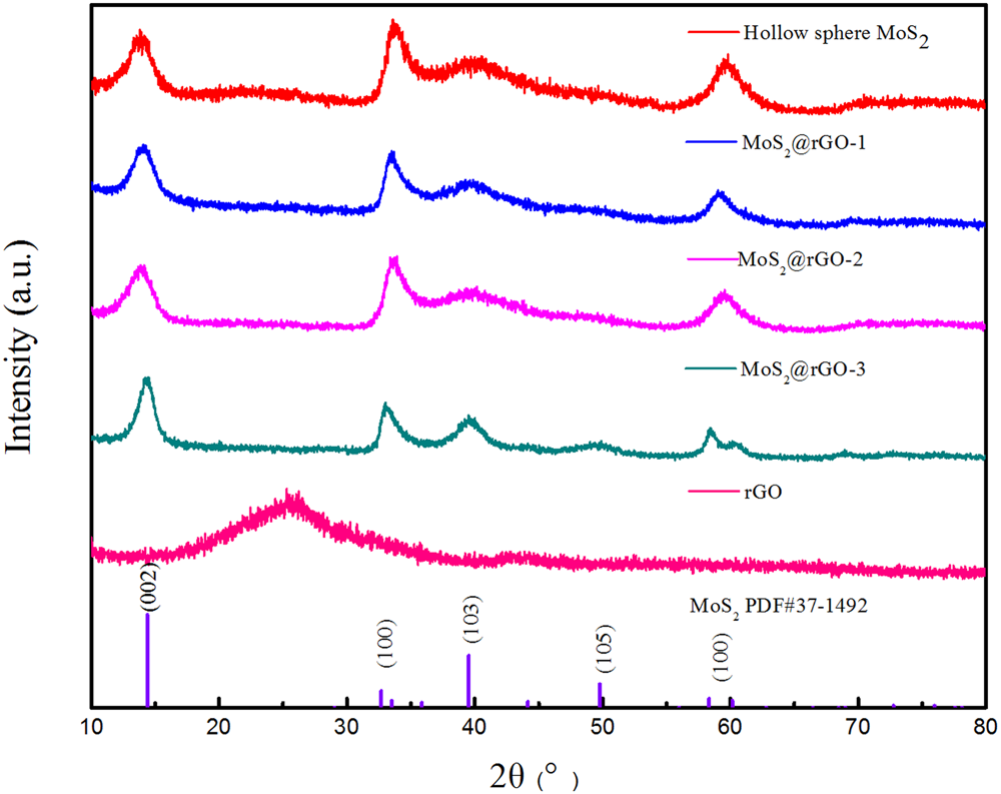
XRD patterns of the prepared rGO, pristine MoS_2_, and MoS_2_@rGO composites.

**Figure 2:**
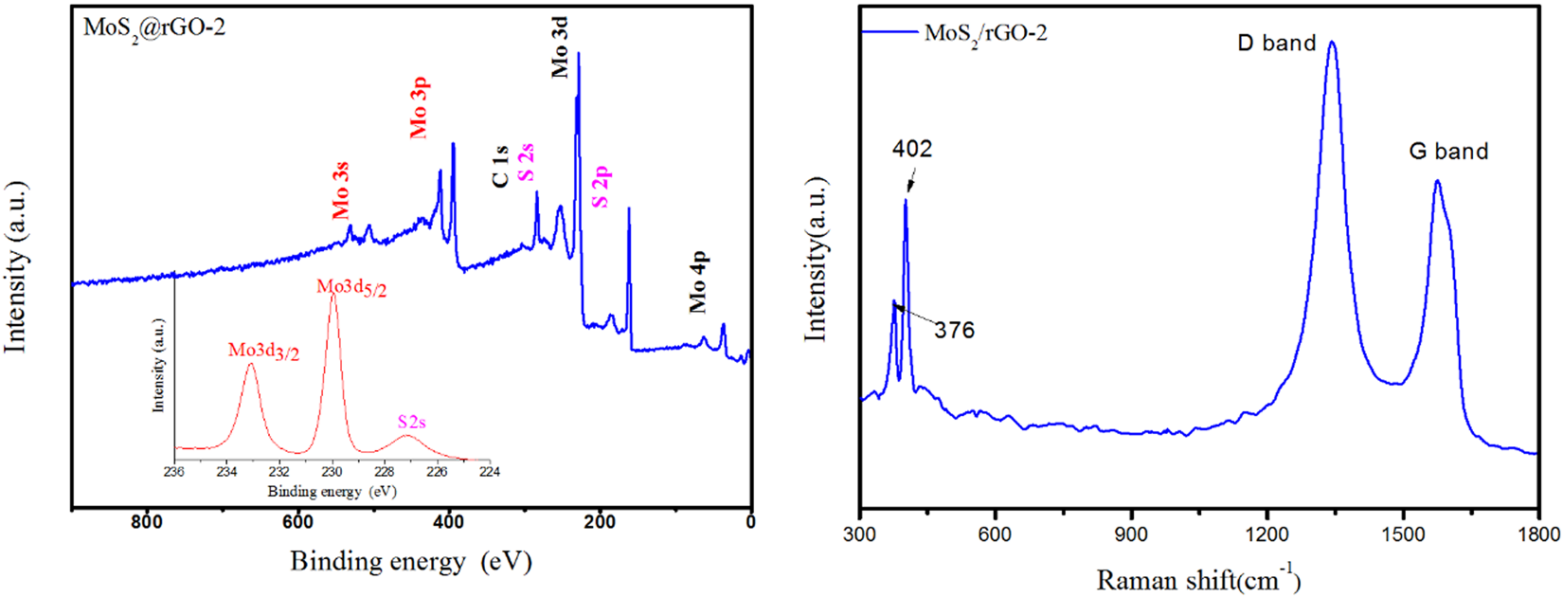
(**a**) XPS spectrum of MoS_2_@rGO-2 composite and the peaks of Mo 3d in the inset graph, (**b**) Raman spectrum of the MoS_2_@rGO-2.

X-ray diffraction spectrometry (XRD) is used to investigate the crystalline phases of the as prepared MoS_2_ and MoS_2_@rGO composites (the composites with different weight ratios of MoS_2_ are named as MoS2@rGO-1,2,3). As shown in Fig. 1, all of the identified peaks belong to the MoS_2_ (JCPDS 37–1492), demonstrating that MoS_2_ is successfully synthesized. Compared to the pristine MoS_2_, the MoS_2_@rGO composites exhibit broadened and less prominent peaks at (002) and (103), suggesting the formation of smaller MoS_2_ crystallite. In addition, the interlayer spacing (*d*_002_ value) of the MoS_2_@rGO composites is calculated to be 0.64 nm, which is larger than that of the pristine MoS_2_ (0.61 nm), implying the expansion in the (002) interlayer of MoS_2_. The expanded interlayer of MoS_2_ can lower the energy barrier of Na^+^ intercalation and facilitate Na^+^ diffusion^32, 33^. Meanwhile, the (002) characteristic broad peak of rGO at about 26° is not observed in the MoS_2_@rGO composites, suggesting the fine distribution and exfoliation of rGO sheets which is beneficial for electronic conducting and high mass loading of MoS_2_. X-ray photoelectron spectroscopy (XPS) measurements are conducted to analyze the composition and chemical states of MoS_2_@rGO composites. The two peaks at 233.0 and 229.9 eV in the inset graph of Fig. 2a are assigned to the binding energies of the Mo 3d_3/2_ and Mo 3d_5/2_ of Mo^4+^. The spectra of S 2p in Fig. S1 (see in supporting information) reveals two peaks at 163.9 and 162.7 eV corresponding to S 2p_1/2_ and S 2p_3/2_, indicating the valence of S is −2. In the meantime, the C1s peak appearing at 285.0 eV (Fig. S1) is related to the binding energy of the sp^2^ C-C bonds of rGO. Raman spectrum of the MoS_2_@rGO-2 composite is also shown in Fig. 2b. The D and G band at 1341 and 1574 cm^−1^ are the feature peaks of rGO. The peaks at 376.1 and 402.0 cm^−1^ are attributed to the planar (${E}_{2g}^{1}$) and out-of-plane (A_1g_) vibrations, respectively, indicating the few stack layered structure of MoS_2_ in rGO^34–36^. The Raman spectra of different MoS_2_@rGO composites are shown in Fig. S2 (ESI). The intensity ratios between D band and G band (ID/IG) of the composites are calculate to be 1.36 (MoS_2_@rGO-1), 1.42 (MoS_2_@rGO-2) and 1.43 (MoS_2_@rGO-3). As is known, the intensity ratio between D band and G band (ID/IG) is an important parameter to evaluate the rGO composites, the higher the ID/IG ratio, the stronger the disorder degree will be. For electrode materials, a higher ID/IG ratio is beneficial for electronic conducting and electrolyte penetration.

The MoS_2_ loading and surface area of the MoS_2_@rGO composites are characterized by the TGA measurement and N_2_ adsorption/desorption isotherm analysis, respectively, which are summarized in Table S1. The contents of MoS_2_ in the composites are determined to be 94.7 wt% (MoS_2_@rGO-1), 90.3 wt% (MoS_2_@rGO-2), and 81.4 wt% (MoS_2_@rGO-3). Corresponding thermogravimetric curves of MoS_2_@rGO composites are shown in Fig. S3. As seen in Table S1, hollow sphere MoS_2_ possess a relative small BET specific surface area of 8.6 m² g^−1^. When compositing with rGO (BET specific surface area is 106.3 m² g^−1^), the BET surface area of the composites increase to 18.9 m² g^−1^ (MoS_2_@rGO-1), 26.3 m² g^−1^ (MoS_2_@rGO-2), and 37.2 m² g^−1^ (MoS_2_@rGO-3). The increased BET surface area may modify the electrode-electrolyte interface and provides fast Na ion diffusion channel. However, the specific capacity of composites may decrease because rGO serves merely as a buffer and conductive skeleton rather than the accommodation of Na ion. Raising the proportion of rGO in the composite is beneficial for the enhanced conductivity but unfavorable for the specific capacity.

**Figure 3:**
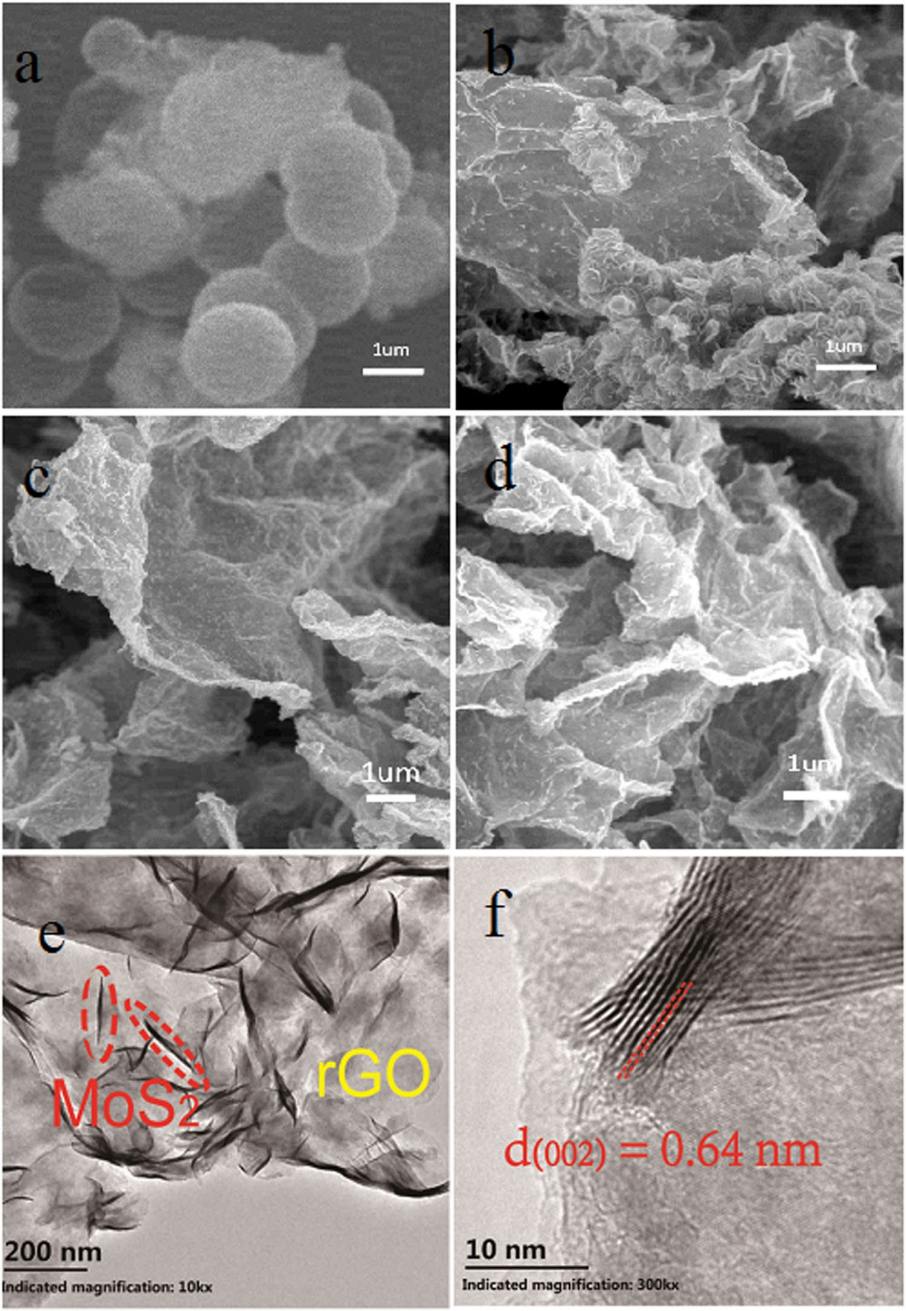
SEM images of the (**a**) pristineMoS_2_ and (**b**–**d**) MoS_2_@rGO2 composites, (**e**,**f**) the TEM images of MoS_2_ nanoflakes on rGO sheets of MoS_2_@rGO-2.

Microstructures of the pristine MoS_2_ and the MoS_2_@rGO composites are further observed by scanning electron microscopy (SEM) and transmission electron microscopy (TEM). The SEM images of products obtained with different concentration of rGO solution are also shown in Fig. 3a–d. As seen, the pristine MoS_2_ in Fig. 3a consists of hollow spherical particles with an average diameter of about 1 um. When rGO is introduced into MoS_2_, hollow spherical structure is effectively inhibited and composite with nanoflake morphology is shown (Fig. 3b). But bulk MoS_2_ still exists for the low ratio of rGO. As the content of rGO increasing, the uniformly distributed MoS_2_ nanoflakes with planar width of 200 nm on rGO sheets are observed in Fig. 3c and d for MoS_2_@rGO-2 and 3. Composites with different rGO contents show significant changes from crumpled and aggregated morphologies to rather flat surfaces with uniformly distribution. The TEM images of MoS_2_@rGO-2 in Fig. 3e and f further verify the lamellar structure of MoS_2_ with an interlayer spacing of 0.64 nm, which agrees with the *d*_002_ of hexagonal MoS_2_ obtained from XRD results. The few-layer MoS_2_ nanoflakes are vertically grown and embedded in the rGO nanosheets, providing sufficient open channels for sodium ion intercalation. Such unique architecture not only enhances the electrical conductivity and stability of active materials, but also favors an increase in the high loading of MoS_2_ in the composites.

**Figure 4:**
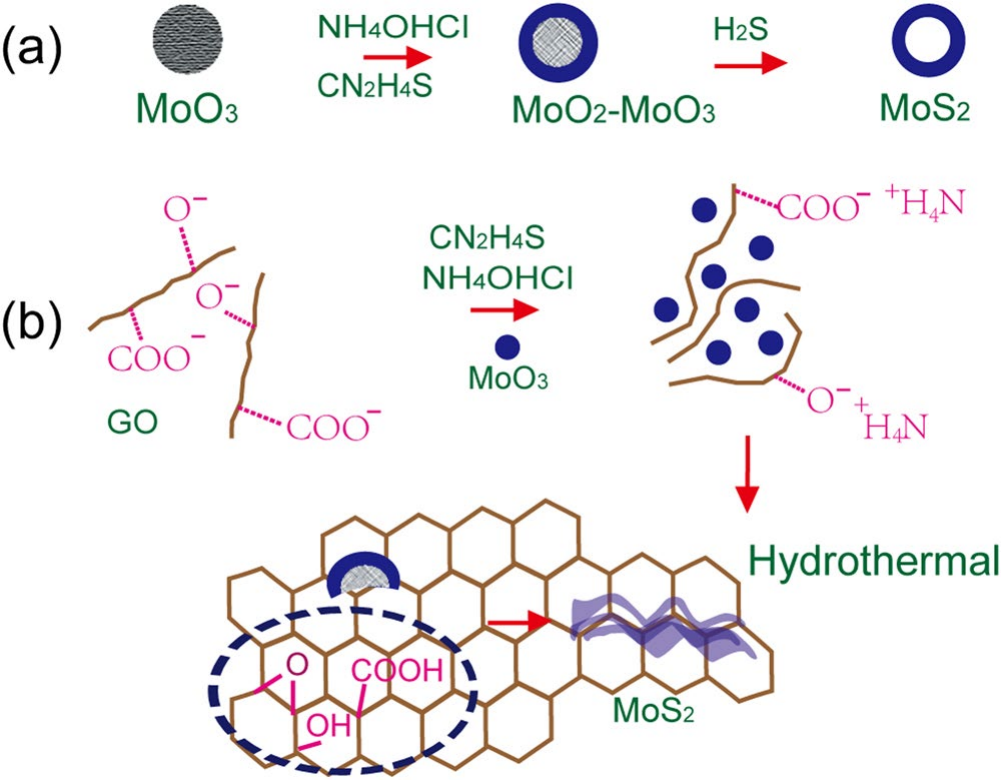
The synthesis of the (**a**) pristine MoS_2_ and (**b**) MoS_2_@rGO composites.

The dramatically different microstructures of the pristine MoS_2_ and MoS_2_@rGO may attribute to the unique synthesis mechanism as displayed in Fig. 4. The formation of pristine MoS_2_ hollow spheres can be explained by Kirkendall effect: First, the MoO_3_ is reduced by NH_2_OH·HCl to form MoO_2_ on the surface (2MoO_3_ + 2NH_2_OH·HCl → 2MoO_2_ + N_2_O + 2HCl + 3H_2_O). In the meantime, NH_2_CSNH_2_ is hydrolyzed to form H_2_S (CS(NH_2_)_2_ + 2H_2_O → CO_2_ + 2NH_3_ + H_2_S). Then, surface MoO_2_ reacts with H_2_S to form MoS_2_ shell outside the MoO_2_-MoO_3_ which serves as the moving boundary in the subsequent reaction. At the last, inner MoO_3_ is reduced to MoO_2_ then react with HCl to form Mo^4+^, which transfer along the formed MoS_2_ boundary and reacting with H_2_S to form MoS_2_ hollow spherical eventually. For MoS_2_@rGO composite, the addition of positive-charged NH_4_^+^ could screen the electrostatic repulsion of GO colloids and the MoO_3_ powders can be uniformly adsorbed onto GO sheets. Owing to the oxygenated functional groups acted as nucleation sites, the MoS_2_ grows gradually perpendicular to the GO skeleton surface and forms nanoflakes as the gradual reaction proceeding during hydrothermal treatment. The unique vertical aligned MoS_2_ nanoflake could maximize the MoS_2_ loading in composites (90.3 wt%) which is much higher than those shown in previous reports.

**Figure 5:**
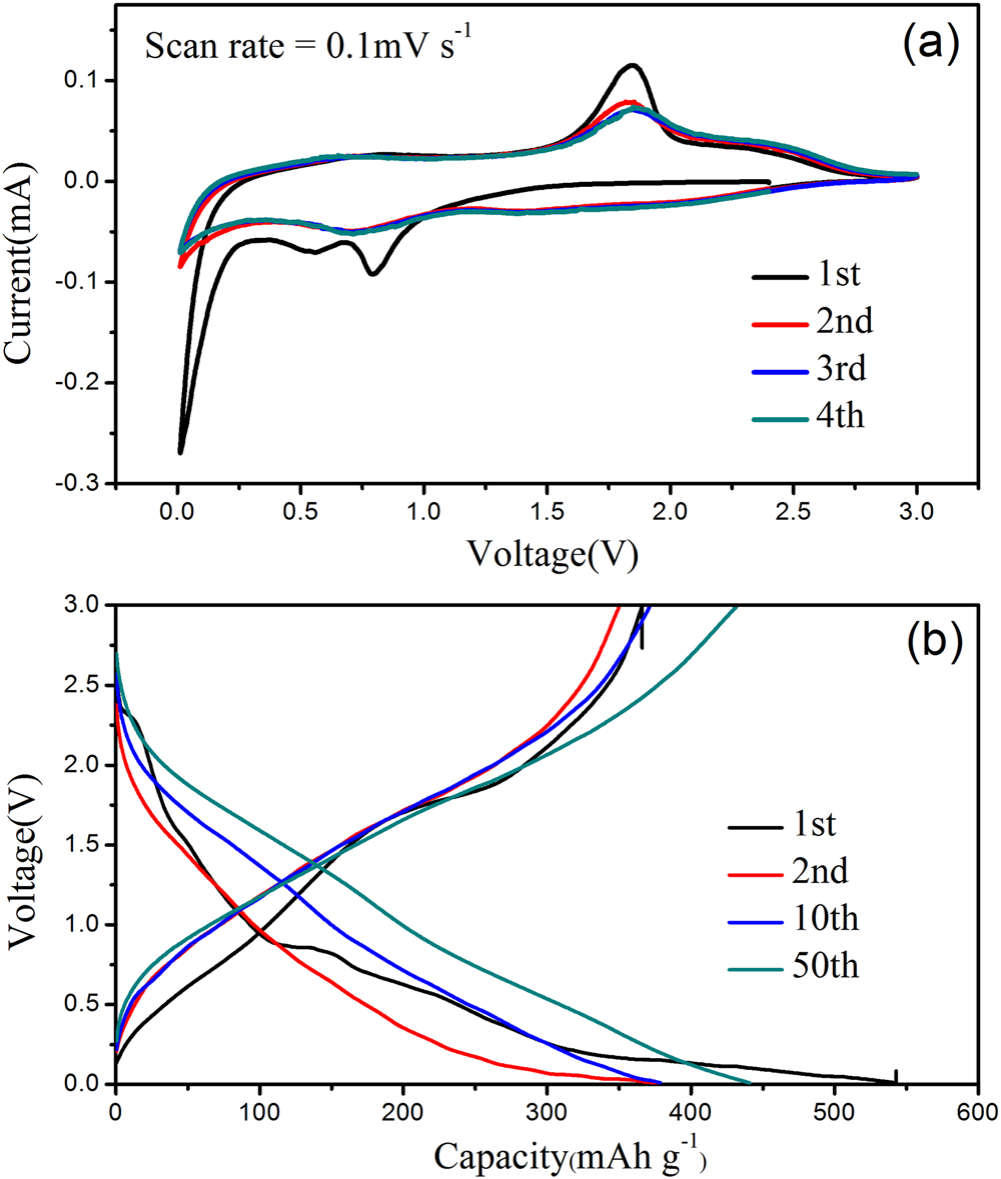
(**a**) CV curves at a potential sweep rate of 0.1 mV s^−1^and (**b**) discharge profiles of the MoS_2_@rGO-2 composite at 0.2 A g^−1^.

The electrochemical properties of MoS_2_ and MoS_2_@rGO composite are characterized by CV and galvanostatic charge-discharge cycling. Figure 5a show the initial four cycles of the MoS_2_@rGO composite in the range of 0–3 V vs. Na^+^/Na at a scan rate of 0.1 mV s^−1^. In the first cycle, three reduction peaks in the sodiation process are observed. The reduction peak at 0.81 Vs. Na^+^/Na is assigned to the Na^+^ insertion into MoS_2_ to form Na_*x*_MoS_2_ (*x* < 2). The following reduction peak at 0.53 V vs. Na^+^/Na is attributed to the further insertion of Na^+^ and at the same time with the formation of solid electrolyte interphase (SEI) layer. The broad peak at 0.1 V vs. Na^+^/Na is related to the conversion of MoS_2_ to form metallic (Mo) embedded in an amorphous Na_2_S matrix. A broad anodic peak observed in the first charging process at about 1.8 V corresponds to the oxidation of the Mo and Na_2_S to MoS_2_. In brief, the sodiation/desodiation of the MoS_2_@rGO composites appears to be based on a two-step reaction process, including an initial insertion process described by MoS_2_ + *x*Na ↔ Na*x*MoS_2_ (*x* < 2) and the following conversion reaction that MoS_2_ converts into Mo and Na_2_S described by Na_*x*_MoS_2_ + (4 − *x*) Na ↔ Mo + 2Na_2_S. The CV curves exhibit no significant variation during the following three cycles, demonstrating the high stability and reversibility of the electrode. The *ex-situ* XRD result (Fig. S4) also identify the compositional changes of the MoS_2_@rGO composites during the first charge/discharge, which is consistent with the CV. The charge-discharge curves of the MoS_2_@rGO composite at a constant current density of 0.2 A g^−1^ are shown in Fig. 5b. The initial discharge curves consist of three plateaus at 1.5–0.8 V, 0.75–0.4 V and 0.4–0.01 V vs. Na^+^/Na, respectively, which are consistent with the CV results. In the subsequent discharge, the plateaus are disappeared and replaced by a long slopes curve as a result of the conversion reaction mechanism, which is in good agreement with previous reports.

**Figure 6:**
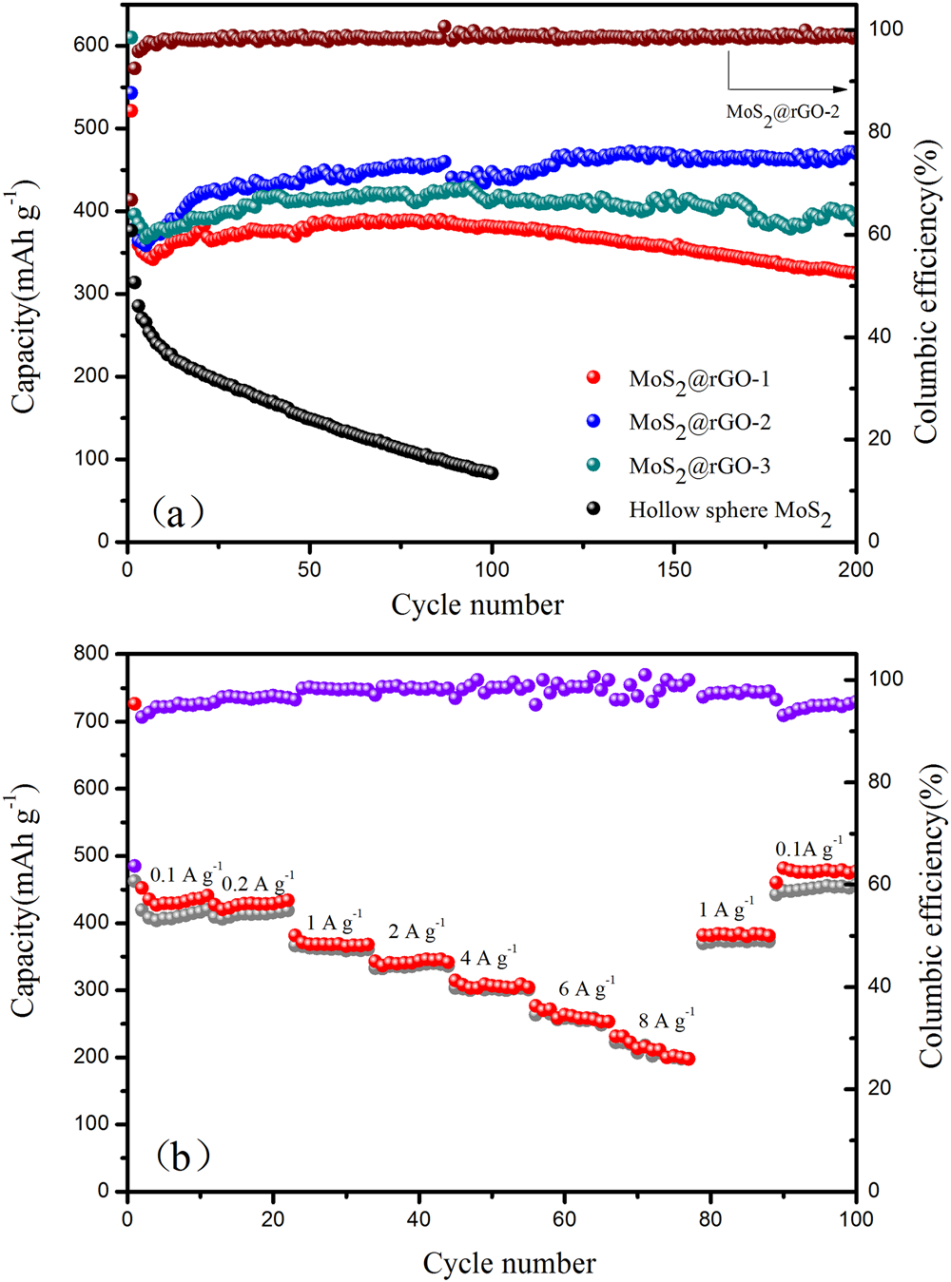
(**a**) The cycle performance of the pristine MoS_2_, MoS_2_/rGO-1,2,3 at 0.2 A g^−1^, (**b**) the rate performance of the MoS_2_@rGO-2 from 0.1 to 8 A g^−1^.

The cycling stability of the pristine MoS_2_ and MoS_2_@rGO composites were further evaluated at 0.2 A g^−1^ as shown in Fig. 6a. The initial discharge capacity of the pristine MoS_2_ is about 470 mAh g^−1^, but drops quickly to 105 mAh g^−1^ after 100 cycles, primarily due to the serious structural collapse and aggregation of the hollow MoS_2_ spheres during the charging/discharging process. On the contrary, MoS_2_@rGO-1,2,3 composites deliver high initial discharge capacities of 521, 543 and 610 mAh g^−1^ with a corresponding first cycle coulombic efficiency of 74.6%, 66.9% and 67.4%, respectively. After the first cycle, the discharge capacity of the composites increased slowly until a stable range of 381, 441 and 412 mAh g^−1^ (50th cycle). MoS_2_@rGO-2 reveals the largest reversible specific discharge capacity. The coulombic efficiency of MoS_2_@rGO composites rise up to 99% rapidly in the initial few cycles (Fig. S5). Moreover, the reversible capacities remain 382, 442, 410 mAh g^−1^ after 100 cycles, nearly 4 times higher than that of the pristine MoS_2_. In the meantime, the electrochemical performance of rGO at a current density of 0.2 A g^−1^ has been evaluated as shown in Fig. S6. The initial discharge capacity of the rGO is about 500 mAh g^−1^, with a low first cycle coulombic efficiency of 13%. The specific discharge capacity drops quickly to 83 mAh g^−1^ at the second cycle, after 100 extended cycles, a low reversible capacity of 54 mA h g^−1^ is maintained. This result further confirms that the specific capacity of MoS_2_@rGO composites mainly come from the MoS_2_. In conclusion, the excellent cycle stability of the MoS_2_@rGO composite may be the results of well developed 3D interconnected nanoflakes structure which benefits fast ion intercalation and provides structural stability. Note that after 300 extended cycles in Fig. S7, a high reversible capacity of 411 mA h g^−1^ is still maintained in the MoS_2_@rGO-2, with a capacity retention of 93.2% based on the reversible capacities of the 50th cycles, corresponding to a capacity decay of 0.027% per cycle. This can be attributed to the higher MoS_2_ loading and more homogeneous dispersion which could effectively inhibit the aggregation of MoS_2_ and accommodate the volume change to maintain the physical stability.

**Figure 7:**
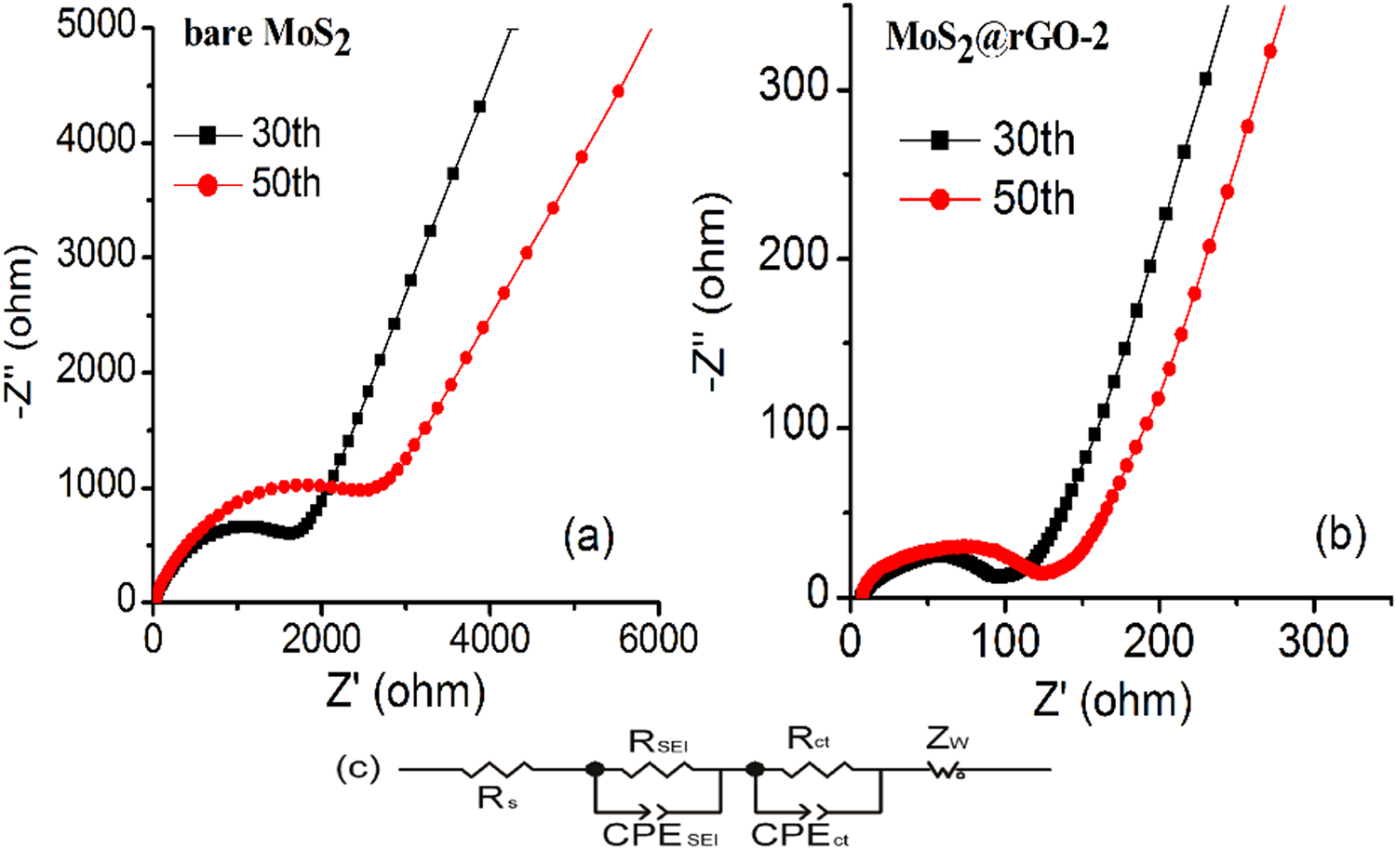
The nyquist plots of the (**a**) pristine MoS_2_, (**b**) MoS_2_@rGO-2, and (**c**) the equivalent circuit for EIS fitting.

Furthermore, the MoS_2_@rGO-2 also demonstrates a very impressive performance at high current density which is shown in Fig. 6b. The reversible discharge capacities of 435, 427, 369, 337, 314 and 258 mAh g^−1^ are obtained at 0.1, 0.2, 1, 2, 4 and 6 A g^−1^, respectively. Even the current density increased by 80 times to as high as 8 A g^−1^, a capacity of 211 mAh g^−1^ is still obtained, with a capacity retention of 48.5%. As the current density decreases back to 0.1 A g^−1^, the reversible capacity switches back with a high value of 477 mAh g^−1^ and keeps stable in the following cycles, indicating an excellent high rate cyclability of the MoS_2_@rGO-2. Apparently, fast kinetics of MoS_2_@rGO are due to the enhanced electrical conductivity combined with an ideal nanoscale architecture consisting of interconnected nanoflakes with short ion diffusion path lengths and good electrolyte accessibility, which is further confirmed by the electrochemical impedance spectroscopy (EIS) measurements. The Nyquist plots of the MoS_2_ and MoS_2_@rGO nanoflakes after different cycles (30^th^ and 50^th^) are displayed in Fig. 7 and Fig. S8 and the fitting results are listed in Table 1. The SEI resistance (*R*_SEI_) and charge transfer resistance (*R*_ct_) of the pristine MoS_2_ increase dramatically during cycling, implying the poor structural stability and sluggish kinetics. For the MoS_2_@rGO nanoflakes, *R*_S_ and *R*_ct_ are much smaller than that of the MoS_2_, and keep almost constant during cycling, indicating a thinner and more stable SEI film favoring rapid Na+ insertion/extraction and fast charge transfer at the electrode/electrolyte interface, which in part explains the high discharge capacity and good rate capability of MoS_2_@rGO nanoflakes that have been observed.

**Table 1:** The fitting results of electrochemical impedance spectroscopy of the pristine MoS_2_ and the MoS_2_@rGO-1,2,3 composite for the 30^th^ and 50^th^ cycles.

Cycle number	*R*_s_ (Ω)		*R*_SEI_ (Ω)		*R*_ct_ (Ω)	
	30^th^	50^th^	30^th^	50^th^	30^th^	50^th^
MoS_2_	20.0	11.1	83.3	2714	1726	6515
MoS_2_@rGO-1	8.8	11.6	10.7	26.7	166.2	247.9
MoS_2_@rGO-2	8.1	6.9	10.7	22.9	67.1	79.5
MoS_2_@rGO-3	5.8	6.2	14.3	16.8	45.3	63.3

## Conclusion

In summary, the ultra-stable MoS_2_@rGO composites as anode materials in SIBs were synthesized via a facile hydrothermal method. Owing to the oxygen functional groups of rGO acted as the nucleation sites, few-layered MoS_2_ nanoflakes are vertically grown and embedded in the rGO nanosheets, which exhibits high specific capacity, outstanding cycle stability and rate capability. The greatly enhanced electrochemical performance of the MoS_2_@rGO nanoflakes can be mainly attributed to the synergistic effect of its unique structural features and high loading of active materials, which not only enhances the electrical conductivity and create more electrochemically active sites, but also alleviate the huge inherent volume change of MoS_2_. Furthermore, the expanded interlayer distance of the MoS_2_ nanosheets benefits fast ion intercalation and provides structural stability, leading to high rate capability and long term cyclability. In addition, this synthesis route with the economical metal oxides as the sources of metal elements may be suitable for the fabrication of other high-loading metal sulfides@rGO composites with outstanding performance in SIBs.

## Methods

### Material synthesis

GO was synthesized in a modified Hummer’s method using the commercial graphite as the original material^37^. MoS_2_@rGO is synthesized by hydrothermal method as follows: 1.0 g MoO_3_, 1.5 g thiocarbamide and 1.5 g NH_4_OHCl were uniformly mixed in 40 ml of DI water via vigorous magnetic stirring. 40 ml of GO suspensions with different concentrations (2, 5, 10 mg ml^−1^) were added to the precursor suspension dropwise and kept stirring vigorously for 20 min. The precursor was then transferred to an autoclave and subjected to a hydrothermal treatment at 180 °C for 48 h. The resultant was rinsed with DI water and ethanol repeatedly via filtration. The precipitate was dried in a vacuum oven at 80 °C overnight. Finally, the as-synthesized samples were annealed at 600 °C for 5 h in a N_2_ atmosphere to reduce the oxygen functional groups of GO and improve the crystallinity of MoS_2_ at the same time. The composites with different weight ratios of MoS_2_ are named as MoS_2_@rGO-1,2,3. The pristine MoS_2_ was synthesized following the same hydrothermal route for comparison.

### Material characterization

The morphology of the prepared composites was characterized by field-emission scanning electron microscopy (FESEM, SIRION200) and high-resolution transmission electron microscopy (HRTEM, JEOL2100). X-ray diffraction (XRD, PANalytical) equipped with a Cu-Kα1 radiation (*λ* = 1.5406 Å) was used to characterize the crystalline structure and interlayer spacing of MoS_2_ in the 2*θ* range of 10–80°. The weight ratios of MoS_2_ in composites were measured by the thermal gravity analysis (TGA, PerkinElmer TGA7) in the atmosphere of nitrogen and oxygen (80:20) at a heating rate of 10 °C min^−1^ in the temperature range of 30–600 °C. The N_2_ adsorption-desorption isotherms were measured using an adsorption unit (Micromeritics, Tristar II 3020) to evaluate the Brunauer-Emmett-Teller (BET) surface area and pore size distribution. Prior to measurement, samples were degassed in a vacuum of 10^−6^ Torr at 150 °C for 12 h to remove the adsorbed moisture. X-ray photoelectron spectroscopy (XPS, Axis Ultra DLD) measurements were performed to analyze the element valence states of MoS_2_@rGO composites with a monochromatic Al-Kα X-ray source. Raman spectra were recorded on a Renishaw Invia spectrometer using Ar^+^ laser of 514.5 nm.

### Electrochemical measurements

The MoS_2_@rGO, Super P, the carboxymethyl cellulose and styrene butadiene rubber (1:1 by weight ratio) were mixed in a mortar at the weight ratio of 8:1:1 to form a homogenous paste and casted on the copper foil. The loading density of the active material was maintained at approximately 1.2 mg cm^−2^. The electrode was dried in a vacuum oven at 120 °C for 12 h. The electrolyte was anhydrous solution of 1 M NaPF_6_ in ethylene carbonate and diethyl carbonate (1:1 in volume ratio). The sodium metal film was used as the counter electrode and the Celgard 2400 was used as the separator. The coin cells (CR2032) were assembled in an argon-filled glove box with the oxygen and moisture levels being less than 0.1 ppm. The galvanostatic charge-discharge tests (LAND, CT2001A) were tested in the potential range of 0.01–3.0 V at 25 °C. Cyclic voltammetry (CV) tests were performed at a scan rate of 0.1 mV s^−1^ in the voltage range of 0.01–3.0 V. Electrochemical impedance spectroscopy (EIS) measurements were performed at an AC amplitude of 5 mV in the frequency range of 10^5^–10^−2^ Hz.

## Electronic supplementary material

Supplementary Information
