# Supplementary material for: MoS2@rGO Nanoflakes as High Performance Anode Materials in Sodium Ion Batteries
Source: Sci Rep. 2017 Aug 11;7:7963. doi: 10.1038/s41598-017-08341-y (PMC5554172; doi:10.1038/s41598-017-08341-y)
Supplement: Supplementary file 1 — Supplementary Information [file 41598_2017_8341_MOESM1_ESM.pdf]

Supporting Information

Title: MoS2@rGO Nanoflakes as High Performance Anode Materials in Sodium Ion Batteries

Ruxing Wang,[†] Shu Gao, [†]Kangli Wang,*,[‡] Min Zhou,[‡] Shijie Cheng,[‡] Kai Jiang,*,[†,‡]

† These authors contributed equally to this work

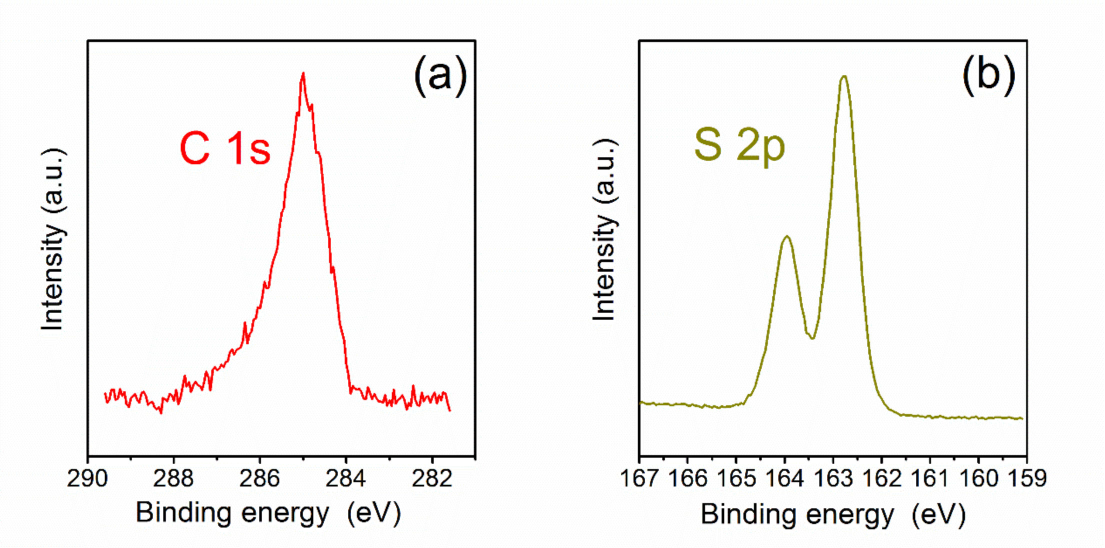

Fig. S1. Fine spectra of C1s (a) and S2p (b) of MoS_2@rGO-2.

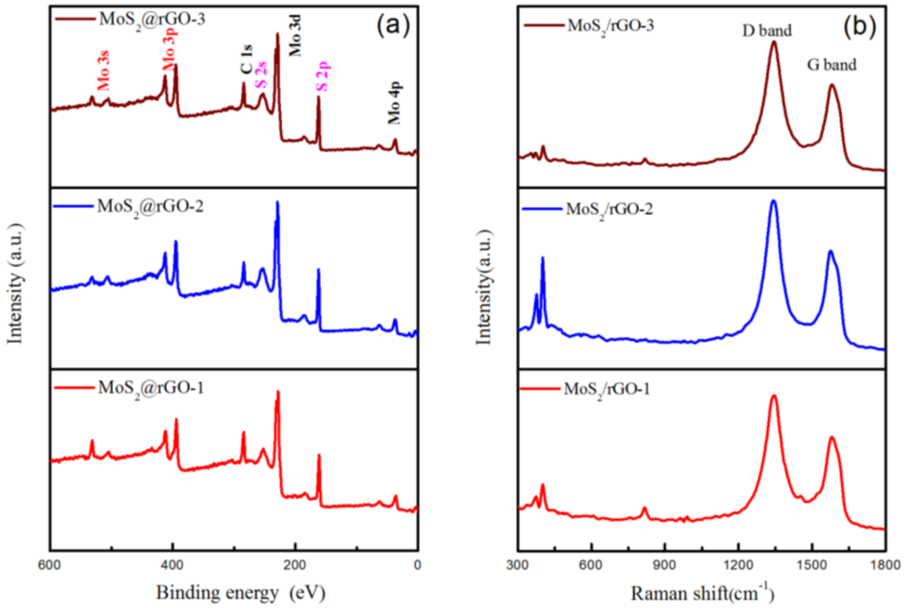

Fig. S2. (a) XPS and (b) Raman spectra comparison of MoS_2@rGO composites.

Table S1. The BET surface areas and mass loading of MoS_2 for samples synthesized.

Sample	MoS_2	MoS_2@rGO-1	MoS_2@rGO-2	MoS_2@rGO-3
BET surface area ($m^2\ g^{-1}$)	8.6	18.9	26.3	37.2
Mass loading of MoS_2 (wt%)	-	94.7	90.3	81.4

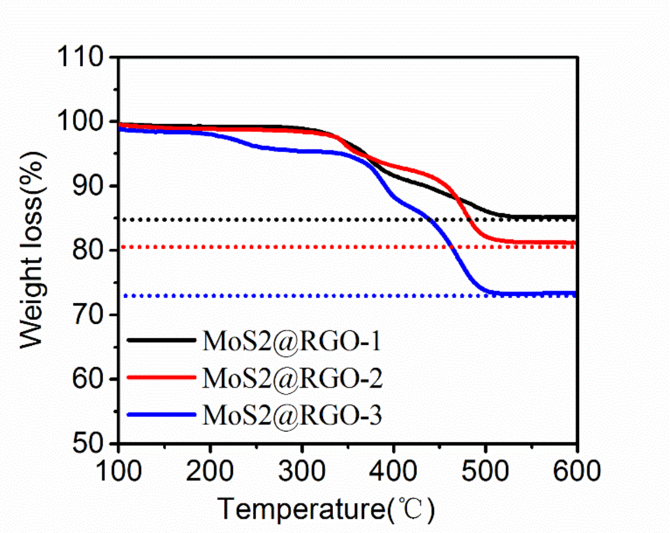

Fig. S3. Thermogravimetric curves of MoS2@rGO composites of 100-600℃.

Ex-situ **XRD analyses of the MoS_2@rGO composites:**

An *ex-situ* XRD analyses are carried out to identify the compositional changes of the MoS_2@rGO composites during the first charge/discharge. As shown in Fig. S4, when discharged to 0.5V, two diffraction peaks corresponding to (100) and (103) crystal planes of MoS_2 still could be observed and some new diffraction peaks appeared in XRD, which imply that $NaMoS_2$ are formed after intercalation. The result confirmed

the intercalation mechanism of lay structured MoS_2. When fully discharged to 0.01V, the MoS_2 peaks disappeared, instead, only a broad peak at about 25° was observed, which are ascribed to the amorphous products of Na_2S and Mo. This result suggests that a conversion reaction takes place during discharging to 0.01V. After first fully charge, diffraction peaks of MoS_2 are emerged out, indicating that Na_2S and Mo are converted to MoS_2 again after a fully charged process. In brief, the sodiation/desodiation mechanism of the MoS_2@rGO composites appears to be based on a two-step reaction process, including an initial insertion process described by $MoS_2 + x\mathrm{Na} \leftrightarrow \mathrm{Na}_x\mathrm{MoS}_2$ ($x < 2$) and followed by a conversion reaction that MoS_2 converts into Mo and Na_2S described by $\mathrm{Na_xMoS_2} + (4\text{-}x)\ \mathrm{Na} \leftrightarrow \mathrm{Mo} + 2\mathrm{Na_2S}$. The XRD results are consistent well with the analyses of CV.

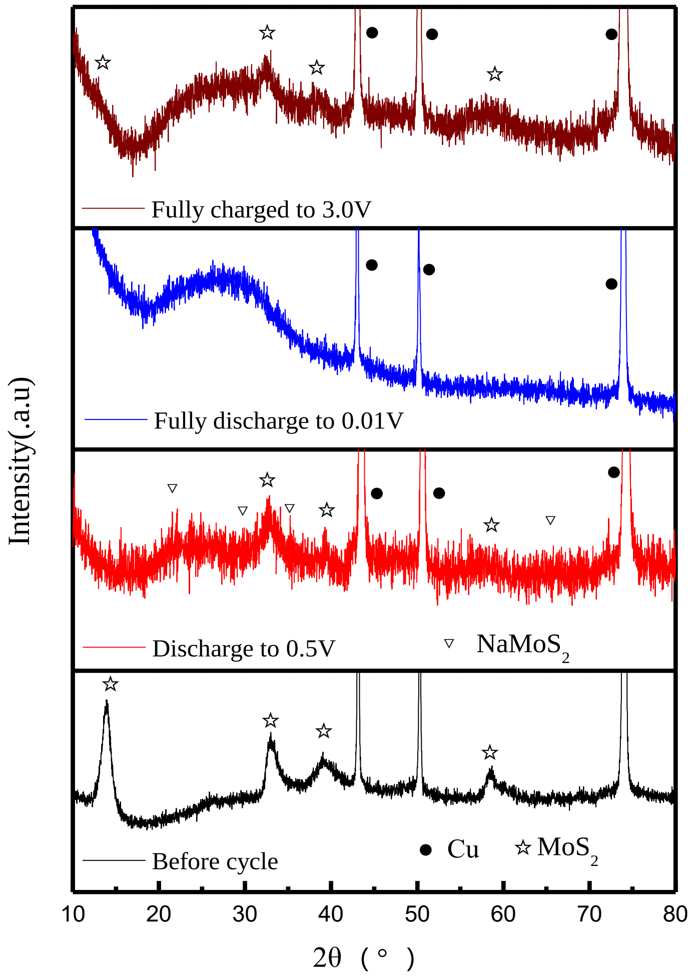

Fig. S4 *Ex situ* XRD patterns of MoS_2@rGO electrodes at different charged/discharged states at the first cycle.

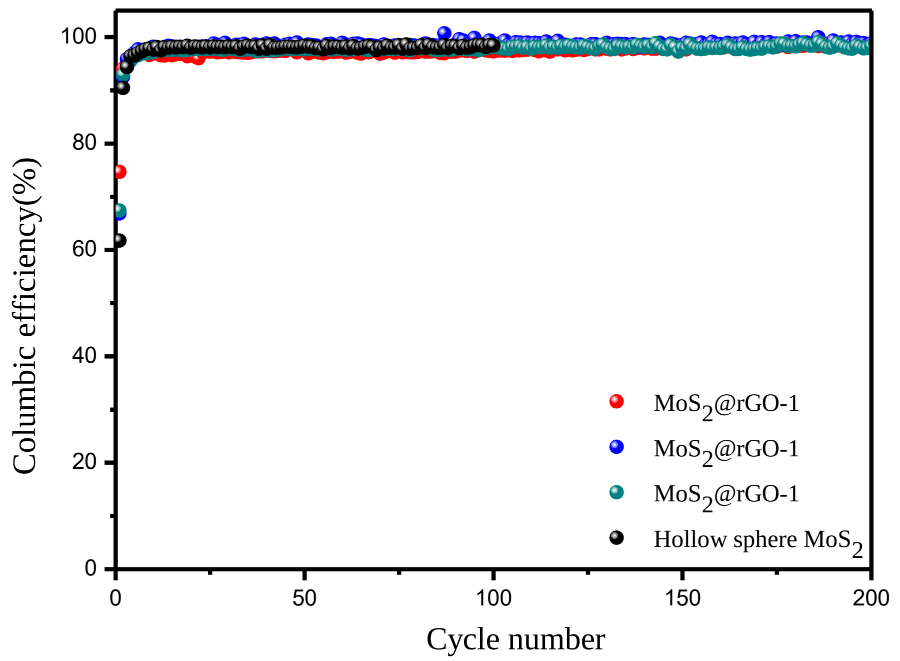

Fig. S5. Coulombic efficiency of the MoS2@rGO-1,2,3 and the hollow sphere MoS2.

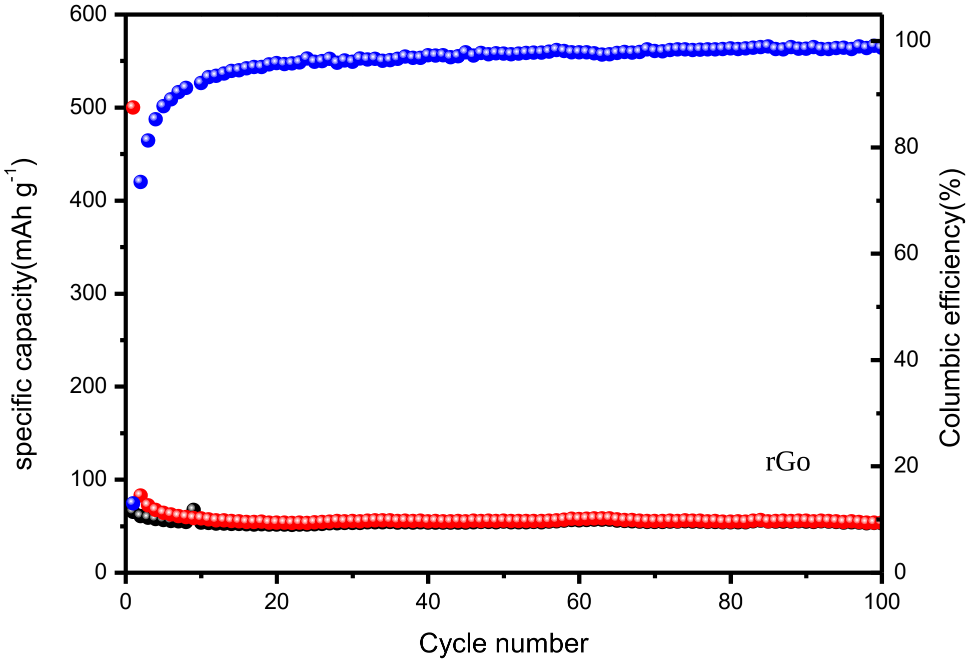

Fig. S6. The electrochemical performance of rGO at a current density of 0.2A g^{-1}.

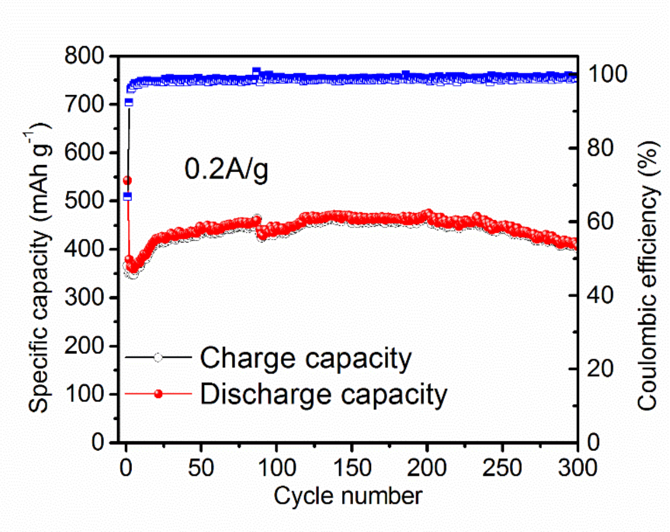

Fig. S7. Battery performance of the MoS_2@rGO-2 for 300 cycles at 0.2 A g^{-1}.

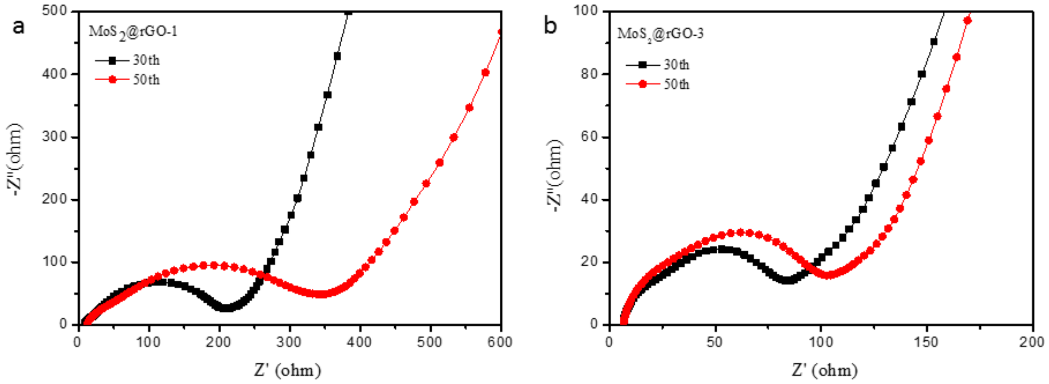

Fig. S8. The nyquist plots of the (a) MoS2@rGO-1 and (b) MoS2@rGO-3

Table S2. Comparison of our prepared MoS_2@reduced graphene oxide (rGO) with previously published MoS_2 SIB anode materials.

**material**	**Molybdenum source**	**MoS_2 load**	**Discharge capacity ($mAh\ g^{-1}$)(current density)**	**Cycle performance (initial capacity retention)**	**Ref.**
MoS_2@reduced graphene oxide (rGO)	MoO_3	90%	440 mA h g^{-1} (200 mA g^{-1})	300 (92.3%)	Thisw ork
MoS_2@reduced graphene oxide (rGO)	Thioacetamide	64%	305 mAh g^{-1} (100 mAg^{-1})	50(nearly no capacity fade)	1
MoS_2/Graphene Composites	phosphomolyb dic acid (PMA)	68.7 %	254 mA h g^{-1} (200 mA g^{-1})	300	2
MoS_2@Graphene	Expanded MoS_2	77 %	313 mAh g^{-1} (100 mAg^{-1})	200 (81%)	3
3D MoS_2@Graphene Microspheres	Ammonium tetrathiomolyb date	83.6 %	480 mAh g^{-1} (200 mAg^{-1})	50(88%)	4
MoS_2@reduced graphene oxide	MoO_3-EDA	92%	420 mAh g^{-1} (100 mAg^{-1})	160	5

References

[1] Qin, W.; Chen, T.; Pan, L., et al., MoS_2-reduced graphene oxide composites via microwave assisted synthesis for sodium ion battery anode with improved capacity and cycling performance. Electrochimica Acta 2015, 153, 55-61.

[2] Xie, X.; Ao, Z.; Su, D., et al., MoS_2/Graphene Composite Anodes with Enhanced Performance for Sodium-Ion Batteries: The Role of the Two-Dimensional Heterointerface. Advanced Functional Materials 2015, 25 (9), 1393-1403.

[3] Wang, Y.-X.; Chou, S.-L.; Wexler, D., et al., High-Performance Sodium-Ion Batteries and Sodium-Ion Pseudocapacitors Based on MoS_2/Graphene Composites. Chemistry – A European Journal 2014, 20 (31), 9607-9612.

[4] Choi, S. H.; Ko, Y. N.; Lee, J.-K., et al., 3D MoS_2–Graphene Microspheres Consisting of Multiple Nanospheres with Superior Sodium Ion Storage Properties. Advanced Functional Materials 2015, 25 (12), 1780-1788.

[5] Che, Z.; Li, Y.; Chen, K.; Wei, M., Hierarchical MoS_2@ RGO nanosheets for high performance sodium storage. Journal of Power Sources 2016, 331, 50-57.
